# Generalisability through local validation: overcoming barriers due to data disparity in healthcare

**DOI:** 10.1186/s12886-021-01992-6

**Published:** 2021-05-21

**Authors:** William Greig Mitchell, Edward Christopher Dee, Leo Anthony Celi

**Affiliations:** 1 Department of Ophthalmology, Massachusetts Eye and Ear Infirmary, Boston, MA USA; 2 Harvard TH Chan School of Public Health, Boston, MA USA; 3 Harvard Medical School, Boston, MA USA; 4 Institute for Medical Engineering and Science, Massachusetts Institute of Technology, Cambridge, MA USA; 5 Department of Pulmonary, Critical Care and Sleep Medicine, Beth Israel Deaconess Medical Centre, Boston, MA USA

**Keywords:** Machine learning, Disparity, Healthcare equity, Ophthalmology

## Abstract

Cho et al. report deep learning model accuracy for tilted myopic disc detection in a South Korean population. Here we explore the importance of generalisability of machine learning (ML) in healthcare, and we emphasise that recurrent underrepresentation of data-poor regions may inadvertently perpetuate global health inequity.

Creating meaningful ML systems is contingent on understanding how, when, and why different ML models work in different settings. While we echo the need for the diversification of ML datasets, such a worthy effort would take time and does not obviate uses of presently available datasets if conclusions are validated and re-calibrated for different groups prior to implementation.

The importance of external ML model validation on diverse populations should be highlighted where possible – especially for models built with single-centre data.

We read with great interest the article describing the application of deep learning to recognize optic disc tilt, and the discussion of its importance in considering ophthalmic measurements, by Cho et al. [1] The rapid evolution of artificial intelligence in ophthalmic image recognition has created unprecedented opportunities for efficient, accurate and cost-effective diagnosis with less human input – of particular value in resource-poor settings where specialist input is relatively scarcer [2]. While we are encouraged by the model accuracy and commend the authors for describing strengths and weaknesses of their study, we would like to highlight a limitation and subsequent area of further exploration that would strengthen the utility of their work: the need to evaluate the generalisability of their model outside their single-centre, paediatric South Korean population. We believe validation of the model developed by Cho et al. on other populations, particularly those lacking local imaging repositories, would be of great value.

Sociodemographic disparities in machine learning (ML) are well described; with recurrent underrepresentation of some populations posing substantial risks of unknown ML biases. Indeed, a recent report noted that 172 countries (totalling 3.5 billion people) have no publicly available ophthalmic imaging datasets [3], profoundly illuminating the possibility of sampling-bias and subsequent poor generalisability in global ML studies. Such disparity in data availability, if left unchecked, may inadvertently perpetuate global health inequity.

Generalisability is *itself* not binary, nuancing issues of sampling bias in clinical applications of ML [4]. A proxy for validity, generalisability is challenged when translating findings across different clinical settings – if not by demographic diversity (as may be the case for Cho et al), then by unique patient-level differences or local clinical idiosyncrasies. Creating clinically useful ML systems is therefore contingent not only upon demographic and clinical generalisability, but also on an understanding of how, when, and why different ML models work in different settings. Although we echo the need for increased diversification of ML datasets, such a worthy effort would take time. The need for increased diversity does not obviate the uses of presently available datasets if conclusions are conscientiously validated and re-calibrated for different populations prior to implementation.

For example, a recent study from India outlined the value of validating ML models on diverse populations; the model, built with relatively homogenous sociodemographic data, was demonstrated to be more broadly-applicable to other populations in disease detection [5]. Indeed, there are myriad publicly-available ophthalmic imaging datasets, whose algorithms could be broadly validated to assess the extent of their value to populations in data-poor regions, which may lack the infrastructure to develop their own repositories [6–13]. As shown by Gulshan and others, validation of algorithms based on inevitably imperfect data can identify when and where these models still hold clinical value. While models may not be universally generalisable [14], identifying populations in which they *are* accurate – and to what degree, and in what circumstances – still holds importance, particularly in allowing countries lacking the infrastructure to build local imaging datasets to still benefit from international ML findings. Investing in the infrastructure for local validation and re-calibration will also lay groundwork for eventual contribution of local data to international repositories, which may be required to enhance local validity of models.

While there are no hard and fast rules as regards the amount of data needed to validate and re-calibrate a model trained on population A before deploying to population B, the process of validation and re-calibration requires certain steps and features [15–17]. Variables in the original model from population A must be present in the dataset from population B. Data from population B for model validation has to be as recent as possible. A target acceptable discrimination and calibration should be set by those who will use the model and those who will be affected by the model. Special attention should be made in evaluating the accuracy in marginalized groups. If the model performance is below the set threshold, then re-calibration is necessary. In general, the number of patients required should be an order of magnitude greater than the number of features in the model. For images, a principal component analysis is performed to determine which image features are important. Another crucial factor in determining the minimum cohort size is the prevalence of the diagnosis for a classification algorithm or the event for a prediction algorithm. The less prevalent a diagnosis or event is, the larger the sample size required.

Although medicine stands to benefit immensely from publicly-available anonymised data and its applications in artificial intelligence [18], building equitable sociodemographic representation in data repositories is crucial. In the meantime, conscientious local validation and re-calibration will elucidate how and when current ML findings can be applied to heterogeneous populations; and may help to ameliorate disparities in access to ML-driven tools. The importance of model validation on other diverse populations should be emphasised where possible, especially for models built with single-centre data.

## Data Availability

Not applicable.

## Abbreviation

ML: Machine learning
